# Lack of guilt, shame, and remorse following weight stigma expression: a real-time assessment pilot study

**DOI:** 10.7717/peerj.10294

**Published:** 2020-12-22

**Authors:** Paige J. Trojanowski, Lauren Breithaupt, Sonakshi Negi, Joseph Wonderlich, Sarah Fischer

**Affiliations:** 1 Department of Psychology, George Mason University, Fairfax, VA, United States of America; 2 Department of Psychiatry, Massachusetts General Hospital and Harvard Medical School, Boston, MA, United States of America

**Keywords:** Weight stigma, Moral emotions, Experience sampling

## Abstract

**Objective:**

Weight stigma is pervasive and is associated with negative health and psychological outcomes. Few studies have examined weight stigma perpetration or the emotions individuals experience after perpetrating weight stigma. This study used experience sampling to explore the nature and frequency of weight stigma behaviors and cognitions and moral emotions (shame, guilt, remorse, pride) in the perpetrator following weight stigma perpetration.

**Methods:**

Participants were college students (*N* = 31, 77.1% female). Participants completed baseline measures of anti-fat attitudes and one week of experience sampling phone prompts assessing: (1) weight stigma behaviors and cognitions and (2) moral emotions. Generalized estimating equation analyses were used to model trajectories of moral emotions after weight stigma events.

**Results:**

Thirty-one participants reported 1,008 weight stigma events over 7.5 days. Feelings of guilt, shame, and remorse decreased after weight stigma perpetration. Individuals also reported feeling less proud after engaging in weight stigma.

**Conclusions:**

Weight stigma occurs frequently as reported by perpetrators. A lack of remorse, guilt, and shame is evident in undergraduates after they express weight stigma; however, individuals in this study also reported feeling less pride after perpetration. This study highlights the need for future studies to explore the expression of weight stigma from the perspective of perpetrators instead of targets. Results highlight the pervasiveness and normative nature of weight stigma perpetration in everyday life and the need to better understand the emotional response following weight stigma perpetration as a potential mechanism of its perpetuation.

## Introduction

Weight stigma is a pervasive social issue that propagates prejudice and stereotyping of individuals who are of higher-weight (Spahlholz et al., 2016; Brewis et al., 2011). Described as the social devaluation or holding negative attitudes of people of higher-weight status (Tomiyama et al., 2018; Tomiyama, 2014), weight stigma may lead to prejudice and discrimination (i.e., unfair or unequal treatment) of higher-weight individuals (Puhl & Heuer, 2009). Weight stigma is highly normative (Puhl & Heuer, 2009). Data suggest that weight stigmatization has actually risen over time. Within the United States, weight bias among adults (Andreyeva, Puhl & Brownell, 2008), medical professionals (Tomiyama et al., 2015), and children (Latner & Stunkard, 2003) appears to be increasing. In addition, implicit attitudes toward groups stigmatized on the basis of sexual orientation, race, and skin-tone appear to show change towards neutrality, while implicit attitudes towards body-weight demonstrate shifts away from neutrality within the past decade (Charlesworth & Banaji, 2019). Furthermore, moralization of weight and the association of larger body size with lack of individual willpower and ‘moral failure,’ contribute to public weight bias (O’Hara & Taylor, 2018; Townend, 2009).

Such trends are concerning given that perceived weight discrimination and weight stigma negatively affect targeted individuals’ health (Hunger & Major, 2015; Hunger et al., 2015). For example, perceived weight discrimination has been linked to psychiatric morbidity and comorbidity (Hatzenbuehler, Keyes & Hasin, 2009; Robinson, Sutin & Daly, 2017; Sutin, Stephan & Terracciano, 2015), and weight stigma has been similarly related to poor psychological health (for a review see Wu & Berry, 2018). Perceived weight discrimination is also associated with poor physical health outcomes, such as cardiovascular risk and increased allostatic load (Udo & Grilo, 2017; Vadiveloo & Mattei, 2017), and chronic medical conditions (for review see Udo, Purcell & Grilo, 2016). It is also linked to decreased health promoting behaviors such as exercise avoidance, healthcare avoidance, and disordered eating behaviors (for reviews see Vartanian & Porter, 2016; Hunger et al., 2015; Puhl & Suh, 2015a; Major, Tomiyama & Hunger, 2018; Phelan et al., 2015).

Moreover, theoretical models propose a cyclic pattern of how the experience of weight stigma begets poor health outcomes (physiologically and psychologically), which promote poor health behaviors and continued experience of weight stigma (Tomiyama et al., 2018; Tomiyama, 2014; Nolan & Eshleman, 2016). Such models highlight the need for larger societal change to reduce the negative health impact of weight stigma. Despite significant evidence suggesting that weight-centric public health campaigns do not improve health and ironically re-inforce stigmatization of higher-weight individuals, policy-level changes are lacking (Hunger, Smith & Tomiyama, 2020; O’Hara & Taylor, 2018). The personal experience of perpetrating weight stigma is poorly understood, which may impede forward progress in changing behavior on an individual level and inform policy-level changes to propagate societal changes.

In sum, weight stigma is common and can result in psychological harm and poor health. Effective interventions are therefore necessary to reduce weight stigma and its downstream effects. Despite this need, interventions aimed at reducing anti-fat prejudice have been largely unsuccessful (Alberga et al., 2016; Daníelsdóttir, O’Brien & Ciao, 2010). In fact, their limited effectiveness may arise in part from a lack of understanding about the process of expressing or perpetrating weight stigma. In particular, little is known about the emotional experience of perpetrating weight stigma. Recent experience sampling studies have explored the experience of weight stigma from the perspective of the target (Vartanian, Pinkus & Smyth, 2014); however, the expression of weight stigma from the standpoint of perpetrators is unclear. This gap in the literature prevents a comprehensive understanding of how frequently individuals engage in weight stigma and how individuals feel after expressing weight stigma.

### Moral emotions

To understand why weight stigma is perpetrated and normalized, it may be helpful to examine how perpetrators of weight stigma feel, specifically with respect to moral emotions, after engaging in this harmful behavior. Moral emotions are emotions that are linked to the interest or welfare of society. They are thought to serve as an “emotional moral barometer,” providing people immediate feedback on the acceptability of behavior (Keyes & Haidt, 2003; Tangney, Stuewig & Mashek, 2007). Negatively valenced “self-conscious” emotions (e.g., shame, guilt, and remorse) often follow deviations from an individual’s moral compass (Scheff & Retzinger, 1991; Tangney, Stuewig & Mashek, 2007; Baumeister, Stillwell & Heatherton, 1994). In contrast, positively valenced moral emotions like pride are experienced following actions that conform to social standards or “doing the right thing” (Scheff & Retzinger, 1991; Tangney, Stuewig & Mashek, 2007).

Importantly, moral emotions, particularly shame and guilt, help to direct behavior following perceived or anticipated moral failure (Gausel & Leach, 2011). Although similar, there is some evidence that shame and guilt may differ in their foci, such that shame is associated with thinking that the self is defective or bad, while guilt is associated with believing that a specific behavior or thought was bad/undesirable (Tangney & Dearing, 2002). As a result, shame has been viewed as motivating avoidance tendencies compared with guilt, which may motivate approach tendencies and efforts to repair wrongdoing (Tangney et al., 1996). However, other research suggests that shame, too, can lead to personal change if one perceives that the moral failure is believed to result from a modifiable self-defect (Gausel & Leach, 2011; Gausel & Brown, 2012; Ferguson et al., 2007). Furthermore, shame and guilt are often highly correlated (Blum, 2008), especially in situations of self-caused wrongdoings, such as perpetrating weight stigma, compared with other-caused wrongdoings (Schmader & Lickel, 2006), and both can motivate behavior change (Lickel et al., 2014). Thus, it is important to understand the roles of shame and guilt in the expression of weight stigma.

Although not widely studied as intervention targets for weight stigma, some evidence from bullying literature implies that guilt and shame may play a role in bullying behavior and lack of bystander intervention (Rieffe et al., 2012; Mazzone, Camodeca & Salmivalli, 2018; Olthof, 2012). For example, similar to the lack of remorse and shame acknowledgement amongst bullies, perpetrators of weight stigma may not experience (or feel less) remorse for their behavior and may engage in shame displacement through other-directed blame (Ahmed & Braithwaite, 2004; Ahmed, 2006). Similarly amongst adults, workplace bullying has been attributed to low shame acknowledgement (Braithwaite, Ahmed & Braithwaite, 2008). Guilt-proneness has also been linked to decreased bullying behavior (Merkin, 2017; Mazzone, Camodeca & Salmivalli, 2016), potentially because of its association with feelings of personal responsibility in situations of offending others, which leads to greater repentance and efforts to make amends (Fisher & Exline, 2006; Merkin, 2017). It is possible that individuals who perpetrate weight stigma, like bullies, do not feel ashamed or guilty after their behavior (Menesini & Camodeca, 2008); however, the relationship between perpetrating weight stigma and the experience of moral emotions is yet unknown.

### Current study

Studies on the expression of weight stigma have typically used self-reports, such as the Antifat Attitudes test (Crandall, 1994). These self-report questionnaires assess weight stigma attitudes but not actual weight stigma perpetration. Additionally, because weight stigma is so normalized, many perpetrators may fail to recall or recognize weight stigma transgressions, given the high degree of social acceptance around weight stigma. Experience sampling circumvents these limitations by collecting data in the natural environment. Only four studies to date have included weight stigma in experience sampling data collection (Carels et al., 2019; Vartanian, Pinkus & Smyth, 2018; Potter, Meadows & Smyth, 2020; Vartanian, Pinkus & Smyth, 2014). This pilot study aimed to explore daily weight stigma expression and trajectories of moral emotions following weight stigma expression in the perpetrators. The study explored the expression of various weight stigma thoughts and behaviors in everyday life, the frequency and types of these thoughts and behaviors, and perpetrators’ negative and positive moral emotions after they had engaged in a weight stigmatizing thought or behavior.

### Hypotheses

The first aim of the present study was to simply describe the frequency and types of weight stigma perpetration from the perpetrators’ perspective as opposed to the recipients’ perspective. The second aim was to examine the trajectories and rates of change of negative (guilt, shame, remorse) and positive (pride) moral emotions after weight stigma perpetration. Due to the normative nature of weight stigma, it was hypothesized that negative moral emotions would not appreciably increase or decrease following weight stigma expression. Based on the limited work examining emotions surrounding expressions of weight stigma (Nguyen & Malti, 2014) and research suggesting that people believe that perpetrating weight stigma motivates people to lose weight (Nolan & Eshleman, 2016; Vartanian & Smyth, 2013), it was hypothesized that positive moral emotions (i.e., pride) would increase after weight stigma events.

## Methods & Materials

### Procedures

Participants from a large, public Mid-Atlantic university were recruited from an online undergraduate research pool and offered course credit for their participation. Participation in the study included an online screen, training phone calls, and answering the phone prompts via a smartphone. Participants did not attend any in-person visits. In order to conceal the true nature of the study, the research was described to prospective participants in an email as a study about thoughts and behaviors individuals may have toward a variety of different groups of people (e.g., the elderly, Mormons, physicians). Participants completed a screening online questionnaire that included weight stigma expression questions and filler questions asking about stigma expression towards the other groups of people. Participants were excluded from the study if they did not have a smartphone. Participants had to report at least 10 instances of weight stigma over the past month (indicating at least occasional recent weight stigma expression) and have a score of 70 or less on the Attitudes Toward Obese Persons questionnaire (ATOP). The ATOP ranges from 0 to 120, with higher scores indicating more positive attitudes towards heavy weight individuals. A score of 70 or less was chosen as a cutoff point to capture individuals with moderate to strong negative attitudes toward individuals who are overweight or obese (Allison, Basile & Yuker, 1991). The original development study for the ATOP demonstrated mean ATOP scores between 63 and 66. Choosing 70 as the cutoff helped to ensure that participants would express average to above-average weight stigma attitudes and engage in some level of weight stigma perpetration. Following the screener, a consent phone call was scheduled where participants were told that they had an equal chance of answering questions about the groups described in the screener during the remainder of the study (i.e., they would be randomly assigned to answer questions in the baseline survey and throughout the week about one of the groups).

Following the consent phone call, participants were sent an email with what they believed to be a randomly assigned link to the baseline questionnaire. In reality, all participants were sent a link to answer questions about their attitudes and behaviors toward higher-weight individuals. Participants were instructed that following completion of the online baseline questionnaire, they would be enrolled in a phone messaging system where they would receive text messages with links to complete a 2–3 min survey of their mood, behavior, and various questions related to the group to which they had been randomly assigned. Following completion of the baseline questionnaire, participants completed one-week of the experience sampling protocol, including a half day of practice, using their personal smartphone to answer questions during six time points between 10:30 AM and 10:00 PM. The six prompts were sent randomly throughout the day with an interval of at least one hour between prompts and an average of four hours between prompts, creating six approximately equal blocks of time. For this pilot study, all experience sampling data were collected using ESMcapture (http://esmcapture.com/). Participants were contacted 2–3 days after being enrolled to ensure they were receiving phone prompts and to answer any questions. Participants received class participation credits for completion of the study. All procedures were approved by the university’s Institutional Review Board (714290-1).

### Materials

#### Baseline measures

Participants completed a baseline questionnaire after sham-randomization (prior to experience sampling data collection) that included an assessment of demographic information such as age, sex, ethnicity/race, height, and weight.

#### Anti-fat attitudes questionnaire

Participants completed a measure of explicit fat bias—the *Anti-fat Attitudes questionnaire* [AFA; (Crandall, 1994)]. The AFA is a 13-item scale designed to assess 3 domains: Dislike (i.e., dislike toward fat people), Fear of Fat (i.e., personal worry about gaining weight or becoming fat), and Willpower (i.e., being fat is due to a lack of willpower). Scores on each item range from 0 (*strongly disagree*) to 9 (*strongly agree*) with higher scores indicating more negative attitudes (current sample *α* = .84). Items are averaged to produce subscale scores, and subscales are averaged to calculate the total score.

#### Attitudes Towards Obese People questionnaire

Participants also completed the *Attitudes Towards Obese People questionnaire* [ATOP; Allison, Basile & Yuker, 1991]. The ATOP scale is a 20-item measure that assesses stereotypical attitudes about obese individuals. Items are rated on a 7-point Likert scale ranging from −3 (*strongly disagree*) to +3 (*strongly agree*) on agreement with specific statements, such as “obese people are not as happy as non-obese people” and “most non-obese people would not want to marry anyone who is obese.” After reverse scoring negatively worded items, possible scores range from 0 to 120, with higher scores indicating more positive attitudes toward obese individuals (current sample *α* = .85).^1^ 1Additional measures that assessed personal experiences with weight-based discrimination, beliefs about appearance, general mood questions, fear of fat/gaining weight, eating pathology, values, and ethnic identity were collected in the baseline survey to examine other research questions.

#### Daily experience sampling measures

The daily phone prompts contained a number of multiple-choice responses and short answer type responses.

#### Weight stigma questions

Participants recorded weight stigmatizing behaviors and cognitions by responding to the following prompt: “Since your last rating, please indicate which of the following you have engaged in (yes/no).” Weight stigmatizing behaviors were taken from previous studies (Puhl & Brownell, 2006; Puhl & Suh, 2015b; Vartanian, Pinkus & Smyth, 2014; Vartanian & Porter, 2016). Participants responded to 23 different behaviors and cognitions, ranging from more overt behaviors such as “suggested a diet to someone because of their weight” and “teased someone because of their weight” to more internal thoughts such as “saw someone overweight and felt bad for them” and “judged someone because of their weight.” The order of the weight stigma questions was randomized at each phone prompt (see Appendix S1 for full list of behaviors). Of note, participants could record more than one weight stigma behavior or cognition at each time point.^2^ 2Participants also responded to questions about basic behaviors in which they engaged since the previous prompt (i.e., watching television, reading, eating, checking social media, exercising) and the length of time doing each activity.

#### Affect assessment

Participants rated their mood using the positive and negative affect scale (International-PANAS-Short Form). They reported on the following 10 items: alert, determined, attentive, inspired, active, upset, afraid, nervous, ashamed, hostile (Watson, Clark & Tellegen, 1988; Karim, Weisz & Rehman, 2011). They rated their current level of each emotion on a scale of 1 (*Not at all*) to 5 (*Extremely*). The item “guilty” was added to assess how feelings of guilt specifically changed after weight stigma perpetration.

The State Shame and Guilt Scale (SSGS) was administered at each phone prompt and was used to inform the development of additional questions related to moral emotions for use in the EMA platform (Marschall, Sanftner & Tangney, 1994). Specifically, students were instructed: “You will be shown a set of statements. Please rate how you feel about yourself right now using the following scale: 1 (*None at all*) to 5 (*Extremely*). The SSGS contains three 5-item subscales assessing guilt (e.g., “I feel remorse, regret”, “I feel bad about something I have done”, “I feel like apologizing, confessing”) shame (e.g., “I feel small”, “I feel like I am a bad person”), and pride (e.g., “I feel good about myself”, “I feel worthwhile, valuable”).

### Data analysis

40 participants were recruited, and all 40 met the minimum weight stigma event requirement over the past month and endorsed moderate to strongly negative attitudes towards higher-weight individuals as measured by the ATOP. After the baseline questionnaire, five of the participants decided not to participate in the experience sampling protocol due to time constraints. A total of 35 participants completed the baseline questionnaire and one-week of phone prompts. Four participants, however, were excluded from the main analyses due to having 3 or fewer responses, leaving 31 participants. Results are based on data from the 31 participants.

#### Frequency and type of weight stigma events

Sample level frequency histograms were used to visualize variability. Sample level frequency histograms reflect the overall number of reported weight stigma events summed over the entire study period. Weight stigma events were divided into behaviors and cognitions.

#### Emotional trajectories following weight stigma

The main goal of the present study was to explore potential changes (or stability) in slope and direction of moral emotions following a weight stigma perpetration. For this reason, a trajectory analysis was used, specifically generalized estimating equation (GEE) models. GEE models were used to examine a linear coefficient, a quadratic coefficient, and a cubic coefficient following weight stigma perpetration in order to determine the trajectories of moral emotions as they changed over time following those behaviors. Trajectory analysis was used specifically to examine the within person rate of change and direction of change in these emotions after weight stigma perpetration. Although time-lagged analyses could be used to compare the mean levels of guilt experienced at one time point to another, this type of analysis would not estimate the rate of change in the emotion as time passes. If separate intercepts were estimated for each event, then the graph could appear to show a precipitous rise or fall in affect immediately at the time of the weight stigma perpetration; however, there would be very few affect recordings immediately surrounding the behavior to support a rise or drop (Engel et al., 2013). GEE models are derivations of the general linear model specifically appropriate for analysis of longitudinal, nested, and repeated measures data that allow for non-normal distributions (Liang & Zeger, 1986).

To assess the relationship between moral emotions and weight stigma events, a dichotomous variable was first created to represent the presence or absence of weight stigma perpetration at each time point. This dichotomous variable was analyzed as the event of interest. Generalized estimating equation (GEE) analyses were used to model each emotion trajectory in the four hours following a weight stigma event. In addition to the single-item ratings of feeling “guilty” and “ashamed,” the trajectories of two items from the SSGS were examined individually because they captured a moral emotion of interest: “I feel remorse, regret” and “I feel proud.” Composite scores for each of the SSGS subscales (guilt, shame, pride) were also calculated for each time point. Trajectories of these composite scores as well as composite scores for PANAS positive and negative affect were examined to validate findings based on the single items and are included in the supplemental results. Models were based upon a gamma distribution (due to significant positive skew) with a log link function and a second-order dependent covariance structure to account for correlation across repeated observations. Linear, quadratic, and cubic components were estimated separately for post-stigma behavior. The linear components indicate whether the initial slopes of the curve closest to the weight stigma event are increasing or decreasing, while the quadratic and cubic components allow deflections from the initial slope and represent accelerations/decelerations in the rate of change when significant. Quadratic and cubic components can only be interpreted if the basic linear component is significant. A common intercept was estimated for post-stigma behavior curves to provide a continuous curve. On days in which multiple weight stigma events were reported, only ratings prior to the second event were included in the analyses to avoid confusion between trajectories of interest (i.e., guilt, shame, remorse, pride).

## Results

### Participants

The sample was primarily female (74.19% female) with a mean age of 19.74 years (*SD* = 2.35, range 18–28) and with a mean BMI, based on self-reported height and weight, of 25.45 kg/m^2^ (*SD* = 5.81, range 17 - 41). 42% of participants had a BMI >25. The racial/ethnic background of the sample was as follows: 67.74% White/Caucasian, 9.68% Asian, 9.68% Hispanic, 6.45% Black/African American, and 6.45% Mixed heritage/unknown. The majority of participants were also born in the United States (87.1%, 27 students).

### EMA compliance

The 31 participants who completed the experience sampling protocol responded for an average of 6.77 days (*SD* = 1.96) with an average of 2.88 responses per day (*SD* = 1.51). The compliance rate averaged 46.4% across participants.

### Baseline weight stigma questionnaires and weight stigma events

Means and standard deviations of baseline weight stigma variables are displayed in Table 1. Correlations between anti-fat attitudes and total weight stigma expression measured via experience sampling also appear in Table 1. Importantly, total weight stigmatizing cognitions and behaviors were significantly correlated with baseline AFA total mean scores (*r* = 0.38, *p* = .04) and the AFA Dislike subscale (*r* = 0.40, *p* = .02), indicating that greater self-reported stigma expression was associated with greater self-reported anti-fat attitudes and particularly with dislike of individuals of higher weight (see Table 1). Total stigma events during EMA did not correlate with ATOP scores. Looking individually at weight stigma cognitions versus weight stigma behaviors, higher frequency of weight stigma cognitions was correlated with less positive attitudes toward higher-weight people (ATOP) (*r* = −.36, *p* = .048) at the trend level, while frequency of weight stigma behaviors was not. In contrast, only weight stigma behaviors had a significant positive correlation with AFA Dislike scores (*r* = .43, *p* = .02) and AFA Total scores (*r* = .39, *p* = .03). AFA Willpower was not significantly correlated with weight stigma perpetration cognitions or behaviors.

**Table 1:** Means, standard deviations, and correlations between variables for subjects in EMA analyses (*N* = 31).

Variable	*M*	*SD*	1.	2.	3.	4.	5.	6.	7.	8.
1. AFA total	3.75	1.52	–							
2. AFA Dislike	1.80	1.53	0.69^*^	–						
3. AFA Fear of Fat	4.34	2.67	0.87^*^	0.42^*^	–					
4. AFA Willpower	5.14	2.07	0.80^*^	0.38^*^	0.52^*^	–				
5. ATOP ^+^	68.00	14.47	−0.42^*^	−0.27	−0.38^*^	−0.29	–			
6. Wt. Stigma Total	32.52	28.77	0.38^*^	0.40^*^	0.25	0.32	−0.31	–		
7. Wt. Stigma Cog.	19.03	16.06	0.33	0.34	0.21	0.29	−.36^*^	0.94^*^	–	
8. Wt. Stigma Behav.	13.48	14.62	0.39^*^	0.43^*^	0.25	0.30	−.22	0.93^*^	0.76^*^	–
9. BMI	25.45	5.81	−0.06	−0.20	0.09	−0.12	0.07	−0.24	−0.24	−0.21

**Notes.**

*M* and *SD* represent mean and standard deviation, respectively.

AFA Antifat Attitudes Questionnaire ATOP Attitudes Toward Obese People Wt Stigma Total Total number of weight stigma perpetrations reported per individual during the experience sampling period. Wt. Stigma Cog. Total number of weight stigma cognitions reported per individual during the experience sampling period. Wt. Stigma Behav. Total number of weight stigma behaviors reported per individual during the experience sampling period BMI Body Mass Index + higher scores indicate more positive attitudes

* *p* < .05.

### Frequency and types of weight stigmatizing perpetration

**Figure 1:**
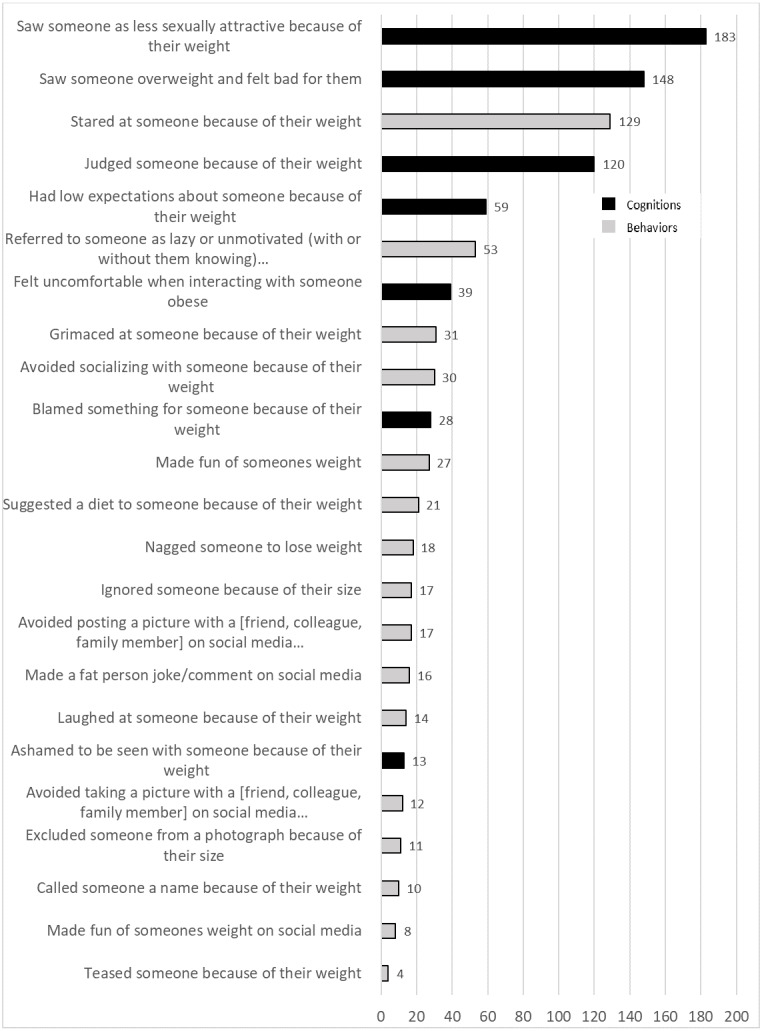
Frequency of specific weight stigma events. Frequency of weight stigma behaviors and cognitions are shown for the 7-day study period*.* Weight stigma event captions ending in “...” were shortened for the graphic but stated “because they were overweight or obese” on the actual experience sampling prompts. Weight stigma behaviors are depicted by gray bars, and weight stigma cognitions are depicted in black bars.

During the 7-day study period, the 31 participants reported perpetrating 1,008 weight stigma behaviors and cognitions. These weight stigma behaviors and cognitions were reported at 249 phone prompts (participants could report multiple weight stigma behaviors and cognitions at each time point). Additionally, the number of expressions reported at each phone prompt ranged from 0–18 with an average of 1.67 expressions (*SD* = 3.00) reported per time point across the 604 responses. Weight stigma events were recorded on all days of the study, and all participants reported perpetrating weight stigma at least once during the study period. Weight stigma events also occurred throughout all time periods of the day; no clear trends were evident. Of the 1,008 events, participants reported 590 weight stigma cognitions and 418 weight stigma behaviors. The frequencies of each type of weight stigma behavior and cognition are depicted in Fig. 1, which is color-coded to illustrate differences between cognitive (black) and behavioral (gray) weight stigma perpetration.

### Trajectories following weight stigma events

**Figure 2:**
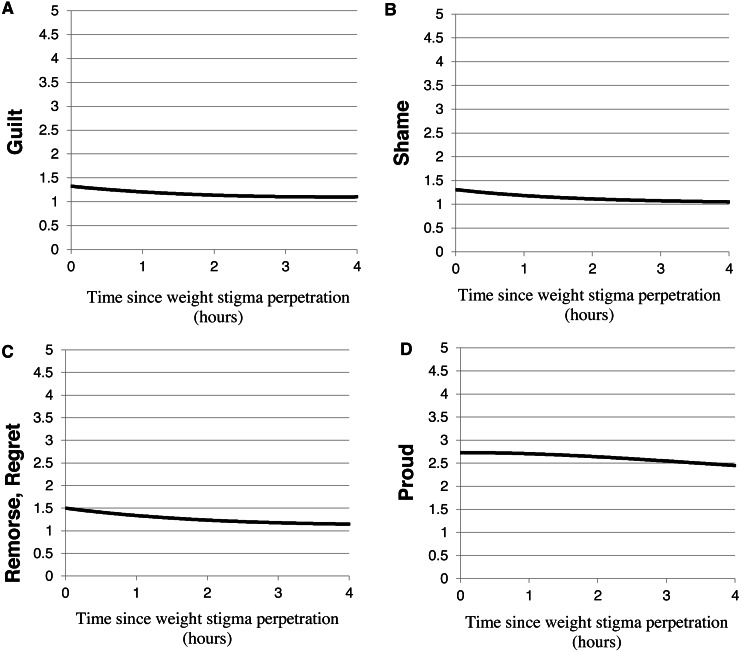
Trajectories of moral emotions. Emotion ratings reported during the experience sampling: (A) “guilty,” (B) “ashamed,” (C) “remorse, regret,” and (D) “proud” following weight stigma perpetration. All moral emotions decreased significantly following weight stigma expression.

GEE analyses indicated that the trajectory of guilt following expression of weight stigma significantly decreased following weight stigma expression (*B* = −.196, *p* = .021, Table 2, Fig. 2A). Similarly, participants reported a significant linear decrease in shame (*B* = −.153, *p* = .007, Table 2, Fig. 2B) and remorse (*B* = −.274, *p* = .002, Table 2, Fig. 2C) following weight stigma perpetration. For guilt, remorse, and shame, the quadratic and cubic components of the trajectory were non-significant, indicating that the rate at which the trajectory was changing over time, or the acceleration, did not change significantly over the 4 hour period following the event.

**Table 2:** General estimating equation (GEE) analyses for single-item assessments of guilt, shame, remorse/regret, and pride following a weight stigma event.

	“Guilty”			“Ashamed”			“Remorse, Regret”			“Proud”		
	*B*	*SE*	*p*	*B*	*SE*	*p*	*B*	*SE*	*p*	*B*	*SE*	*p*
Intercept	**.282**	**.062**	**<.001**	**.269**	**.045**	**<.001**	**.405**	**.084**	**<.001**	**1.004**	**.057**	**<.001**
Hours* Wt. Stig. Event(linear)	**−.196**	**.085**	**.021**	**−.153**	**.057**	**.007**	**−.274**	**.090**	**.002**	**−.169**	**.079**	**.033**
Hours^2^ * Wt. Stig. Event(quadratic)	.013	.012	.279	.023	.015	.122	.001	.012	.947	**−.037**	**.015**	**.016**
Hours^3^* Wt. Stig. Event (cubic)	−.001	.001	.122	**−.002**	**.001**	**.044**	**−.002**	**.001**	**.037**	.000	.001	.999

**Notes.**

Wt. Stig. Weight Stigma

Significant (*p* < .05) estimates of the linear, quadratic, and cubic components of the trajectories appear in bold. “Guilty” and “ashamed” were single items assessing mood, and “I feel remorse, regret” and “I feel proud” were single items from the State Shame and Guilt Scale (SSGS).

Similar to the trajectory of remorse, the trajectory of feeling proud after perpetrating weight stigma also significantly decreased following weight stigma events. Participants reported a significant linear decrease in pride (*B* = −.169, *p* = .033, Table 2, Fig. 2D). The quadratic component of the trajectory was also significant (*B* = −.037, *p* = .016, Table 2), which indicates that the rate at which pride was decreasing after the event accelerated over 4 h after the weight stigma event.

Analyzing the composite scores of the SSGS subscales for guilt, shame, and pride (tables provided in Supplemental Files), showed that guilt decreased significantly following weight stigma perpetration (*B* = −.247, *p* = .01), shame decreased following weight stigma perpetration (*B* = −.096, *p* = .05) at a trend level, and pride did not fluctuate significantly following weight stigma perpetration (*B* = −.059, *p* = .17). Negative affect, measured as a composite of PANAS items, decreased significantly following weight stigma perpetration (*B* = −.216, *p* = .01) as did positive affect (*B* = −.089, *p* = .01) (tables provided in Supplemental Files).

## Discussion

In the first pilot study exploring weight stigma expression from the perpetrators’ perspective in a natural environment, weight stigmatizing behaviors or cognitions were reported every day, by all participants, throughout all time periods of the day. Importantly, these findings are consistent with experience sampling and daily diary studies conducted from the perspective of targets of weight stigma (Seacat, Dougal & Roy, 2016; Vartanian, Pinkus & Smyth, 2018; Potter, Meadows & Smyth, 2020; Vartanian, Pinkus & Smyth, 2014) as well as qualitative studies of experienced weight stigma by higher-weight individuals (Pickett & Cunningham, 2018; Thomas et al., 2008). For example, one experience sampling study of higher-weight women reported an average of three weight stigmatizing events per day, with over 1,000 weight stigma events over a 7-day period (Seacat, Dougal & Roy, 2016), strikingly similar to the present results quantifying stigma perpetration. The results of this pilot study reinforce findings from studies from the targets’ perspective by demonstrating that, among young adult college students, perpetrators readily admit to high levels of weight stigma expression, helping to further illustrate the pervasive and normative nature of weight stigma. Results suggest that experience sampling studies may better capture daily weight stigma perpetration more accurately than retrospective recall, which often results in less frequent reporting of weight stigma events (e.g., Sarwer et al., 2008). Contrary to hypotheses that moral emotions would not appreciably increase or decrease following weight stigma expression, negative moral emotions (guilt, shame, remorse) decreased after individuals expressed weight stigma. Interestingly, trajectories of pride also decreased after expressing weight stigma. Taken together, weight stigma is widespread, and individuals feel negative moral emotions, particularly guilt, shame, and remorse, to a *lesser degree* following weight stigma expression.

People who believe that weight is a direct result of self-control and willpower fail to recognize the many factors that contribute to weight status and hold individuals of higher-weight unduly responsible for their size, leading them to develop negative views of higher-weight individuals (e.g., they are lazy) and blame them for their weight status (Puhl & Brownell, 2003; Crandall & Martinez, 1996). Cross-sectional studies demonstrate that beliefs about the controllability of weight often correlate with anti-fat attitudes (Puhl et al., 2015; Hansson & Rasmussen, 2014; Crandall, 1994). This relationship is particularly true in societies with strong cultural values of thinness. This theory of weight-stigmatization is known as the Attribution-Value model (Crandall et al., 2001). In this sample, however, weight stigma perpetration was not correlated with controllability beliefs (i.e., AFA Willpower). Authors cannot be sure why this result was observed, but multiple hypotheses are offered. It should be noted, however, that these explanations are post hoc and were not directly tested in our study. These may represent hypotheses for future research. First, almost half of the participants were higher-weight, increasing the likelihood that they had insight into the lack of controllability of weight, potentially making them more understanding and sympathetic, thereby weakening the relationship between controllability beliefs and weight stigma perpetration. Second, it is possible that momentary assessment of weight stigma is capturing something different from general self-reports of anti-fat attitudes. Individuals may be less likely to reflect on causal and controllability beliefs in-the-moment when perpetrating weight stigma compared with when they are completing questionnaires that prime them to reflect on their attitudes about weight controllability, thus, leading to the lack of correlation between causal weight attributions and weight stigma perpetration frequency. Third, it might be that beliefs about controllability do not actually drive weight stigma but that people use controllability as a post-hoc rationalization for their stigmatizing thoughts and behaviors. For example, some research suggests that disgust could be responsible for a visceral reaction to higher-weight individuals (Vartanian, Trewartha & Vanman, 2016), leading to weight stigma thoughts and behaviors, which are then justified by the perpetrator using controllability beliefs. In a similar vein, results did support a correlation between *dislike* of higher-weight and overall frequency of weight stigma behaviors, suggesting that dislike may be more related to blatant weight stigma perpetration on a daily level than beliefs about controllability. Last, the present study had a relatively small sample size, which might be the most logical reason for the absence of this correlation. Future studies with larger samples are needed to clarify the relationship between weight controllability beliefs and daily perpetrated weight stigma.

Prior research on weight stigma has shown that society does not always perceive perpetuation of weight stigma as problematic or unacceptable (Crandall & Martinez, 1996; Vartanian & Smyth, 2013). Likewise, the most striking finding of the present study was that participants felt *less* guilt, shame, and remorse after perpetrating weight stigma. This finding may result in part because society shames higher-weight individuals, reducing feelings of personal fault in those who perpetrate weight stigma. Additionally, other system-justifying ideologies, for example, just-world beliefs, Protestant work ethic, and social dominance theory, may play a role in weight-stigmatization and help to further explain these results. These “justification ideologies” essentially protect individuals who perpetrate weight stigma from feeling guilty and have been linked to anti-fat attitudes. For example, people who have strong just world beliefs assume that the world is ultimately fair and that people get what they deserve (Lerner, 1980). When people believe in a just world, by extension, they may believe that higher-weight individuals act in a way that leads to their higher-weight status, and therefore deserve society’s negative treatment (Crandall, 1994; Ebneter, Latner & O’Brien, 2011). Relatedly, weight stigmatizing beliefs are also associated with believing that if someone works hard with determination and persistence, good things or success will result–known as the Protestant work ethic (Crandall, 1994; Puhl & Brownell, 2003). If someone assumes that an individual of higher-weight has simply not worked hard enough to achieve the cultural ideal of thinness and is lazy or lacks self-control, then they may blame the individual for their plight. It is unlikely that they would feel bad after perpetrating weight stigma, and moreover, they may perversely believe that their stigmatizing behaviors will motivate behavioral change in higher-weight people (Vartanian & Smyth, 2013). As such, they may feel that they are doing right by society, thereby decreasing their experience of negative emotions. Finally, social-dominance orientation (Pratto et al., 1994), or the desire for hierarchical social structure and belief that certain groups are more deserving of higher status and even supposed to dominate other groups, has been linked to weight controllability beliefs and anti-fat bias (Crandall, 1994; O’Brien et al., 2013; Magallares, 2014). If one feels that the in-group (e.g., lower-weight individuals) deserves superior treatment to the out-group (e.g., higher-weight individuals), they may feel that they are righting a social wrong by stigmatizing someone of the outgroup, thus, maintaining the social hierarchy and promoting a decrease in negative affect (e.g., guilt, shame). Future studies should thus examine how these ideological beliefs relate to daily weight stigma perpetration.

Pride however, also decreased after weight stigma expression. It is possible that people may have felt less pride because they were interacting with individuals who are typically stigmatized by others in society, and thus, fearing “stigma by association” (Hebl & Mannix, 2003), as opposed to feeling less pride because of their own behavior. Alternatively, individuals may recognize on some level that their stigmatizing thoughts and behaviors, which are based on superficial characteristics, are not something about which to be proud, reflecting small decreases in pride, but may not experience concomitant increases in guilt as might be expected. Additionally, people experience different types of pride (e.g., authentic and hubristic/narcissistic), and a single-item rating may not have adequately distinguished between these types of pride (Carver & Johnson, 2010). Future studies may benefit from more detailed examination of pride following weight stigma perpetration.

### Limitations & future directions

This pilot study contributes to a limited body of work using real-time assessment of weight stigma, however, several limitations should be noted. Foremost, participant compliance rate was significantly lower than in other studies using experience sampling. The reason for the low compliance rate in the present study is unknown, but based on past publications from the authors’ own lab where compliance rates were higher (>80%) across participants (Fischer et al., 2017), differences in data collection are highlighted. Participants in this study were provided with class participation credit at one time point for study completion. In past studies from this lab (e.g., Fischer et al., 2017) where compliance rates have been higher, researchers provided incentives for completing additional prompts, in addition to completion incentives. In the current pilot study, participant compliance was not monitored routinely, and participants were not contacted when their response rates fell below ideal compliance rates (e.g., below 80%). Future studies may benefit from additional safeguards to ensure participant compliance, such as increasing communication frequency with participants, offering extra class participation credits based on the number of prompts answered, or decreasing participant burden by decreasing the number of prompts.

Experience sampling methodology has additional limitations that are inherent to correlational research. Due to the nature of experience sampling, in which participants are asked to report on multiple events and emotions periodically throughout the day, it is not possible to isolate weight stigmatizing events from other events that may have occurred during the same time period in which participants were responding to prompts. The only way to state that engaging in a weight stigma event *caused* a decrease in guilt and other emotions is to conduct an experimental study in which engagement in a weight stigma event is manipulated. These data show that the trajectory of guilt and other moral emotions statistically significantly decreased following these events, but it is always possible that this decrease may have been due to other behaviors or events that occurred at the same time. However, other behaviors that may cause decreases in moral emotions would likely need to occur at the same rate as weight stigmatizing behaviors during the same signaled prompt period in order for those behaviors to have a statistically significant impact on the emotion trajectory. It is possible that the use of signal-contingent prompting (e.g., asking participants to initiate data collection when they engage in an event) may increase confidence in concluding that moral emotions changed as a result of that specific event. However, given the pervasive nature of weight stigma documented in this study and normalization of weight stigma, it is also possible that participants would not have self-initiated data collection for a signal-contingent responding protocol. Last, it is possible that participants were over-reporting their weight stigma cognitions and behaviors due to reactivity to the experience sampling protocol and increased monitoring of thoughts and behaviors. Reported cognitions and behaviors could additionally be related to the same instance. For example, students may have reported judging someone due to their weight, finding them less sexually attractive, and excluding them from a picture due to their weight all at the same time. Future studies should ask participants to distinguish if reported weight stigma applied to one or multiple scenarios since their previous prompt.

Further, the data for the current study were collected from college students. While weight stigma is prevalent on college campuses (Puhl & Suh, 2015b), future studies should explore daily weight stigma expression in other environments. The sample size of the current study was also fairly small (although there were a large number of weight stigma events) and included very few males. The small sample size also precluded examining factors such as race, country of origin, gender, or BMI, or differences between weight stigma cognitions and behaviors as moderators of weight-stigma expression. Although the sample was on average higher-weight, BMI was not correlated with weight stigma perpetration or explicit anti-fat bias, suggesting that this sample of college students appeared to engage in similar patterns of weight stigma perpetration regardless of personal body size; however, future studies should examine weight stigma perpetration differences based on BMI in greater detail given the small sample size.

The present sample was fairly diverse with 34% of the sample being non-White. Cultural or racial differences in body ideals are widely recognized (Grogan, 2016) and may influence people’s attitudes toward people who are overweight or obese as well as their engagement in weight stigma. For example, White women have been found to express more stigmatizing attitudes toward other women, especially White women, compared with African American women. African American women also rated depictions of African American women who were larger bodied significantly less negatively (Hebl & Heatherton, 1998). Similar results have been found in adolescents (Gray et al., 2011). Replication in larger samples would allow for examination of the potential moderating effects of race, ethnicity, BMI, and gender on weight stigma expression.

Future research addressing these limitations should be conducted in order to improve confidence in the current results and better understand weight stigmatizing behavior across multiple contexts and in different cultures. Subsequent studies should also examine what characteristics make people most likely to exhibit weight stigma and less likely to experience guilt following its expression. Interestingly, some research suggests that weight status does not affect people’s stigmatization of obesity, suggesting that higher-weight individuals may be just as likely to engage in weight stigma as lower-weight individuals (Latner, Stunkard & Wilson, 2005). A better understanding of who expresses weight stigma, what motivates them to do so, and what maintains this behavior (e.g., lack of guilt following weight stigma expression) could help to inform intervention and policy development aimed at reducing this harmful and prevalent phenomenon.

## Conclusions

Young adults frequently engage in weight stigma expression, both behaviorally and cognitively on a daily level–a discouraging finding given the harmful effects experienced by targets of weight stigma. In this small pilot study, expressing weight stigma did not evoke moral self-conscious emotions of shame, guilt, or remorse in the weight stigma perpetrator. In fact, individuals reported feeling less shame, guilt, and remorse after expressing weight stigma. These results highlight the need to continue studying expression of weight stigma and moral emotions surrounding the behavior in larger, more representative samples. A clear need to combat the normative nature of weight stigma exists. The authors look forward to, and encourage, future research targeting weight stigma expression.

##  Supplemental Information

10.7717/peerj.10294/supp-1 Appendix S1 Weight Stigma Behaviors and Cognitions Assessed via EMA

10.7717/peerj.10294/supp-2 Table S1 General estimating equation (GEE) analyses for SSGS guilt, shame, and pride subscales following a weight stigma event Note. Wt. Stig. Weight Stigma. Significant (*p* <.05) estimates of the linear, quadratic, and cubic components of the trajectories appear in bold. *indicates a trend (<.10) toward statistical significance.

10.7717/peerj.10294/supp-3 Table S2 General estimating equation (GEE) analyses for the positive affect and negative affect PANAS subscales following a weight stigma event. Note. Wt. Stig. Weight Stigma. Significant (*p* < .05) estimates of the linear, quadratic, and cubic components of the trajectories appear in bold.

10.7717/peerj.10294/supp-4 Supplemental Information 1 Codebook for baseline and experience sampling variables

10.7717/peerj.10294/supp-5 Supplemental Information 2 Baseline variables The data collected at baseline before participants began the experience sampling protocol and consists of demographic information and baseline questionnaires.

10.7717/peerj.10294/supp-6 Supplemental Information 3 Experience Sampling Data The experience sampling data that was analyzed (i.e., weight stigma behaviors and cognitions and mood ratings).

## Additional Information and Declarations

### Data Availability

The following information was supplied regarding data availability: The raw data are available in the Supplemental Files.
