# Supplementary material for: Lack of guilt, shame, and remorse following weight stigma expression: a real-time assessment pilot study
Source: PeerJ. 2020 Dec 22;8:e10294. doi: 10.7717/peerj.10294 (PMC7761191; doi:10.7717/peerj.10294)
Supplement: Appendix S1 [file peerj-08-10294-s001.docx]

Appendix A

Below is the full list of weight stigma behaviors that participants reported at each EMA time point. Participants were instructed as follows: “You will be shown a series of questions about behaviors you may have engaged in. Please respond YES if you have engaged in the behavior and NO if you have not engaged in the behavior SINCE YOUR LAST RATING.”

1. Excluded someone from a photograph because of their size.
2. Avoided posting a picture with a [friend, colleague, family member] on social media because they were over-weight or obese.
3. Avoided taking a picture with a [friend, colleague, family member] on social media because they were over-weight or obese.
4. Referred to someone as lazy or unmotivated (with or without them knowing) because of their size.
5. Ignored someone because of their size.
6. Saw someone overweight and felt bad for them.
7. Avoided socializing with someone because of their weight.
8. Felt uncomfortable when interacting with someone obese.
9. Saw someone as less sexually attractive because of their weight.
10. Ashamed to be seen with someone because of their weight.
11. Made fun of someone’s weight on social media.
12. Made a fat person joke/comment on social media.
13. Made fun of someone’s weight.
14. Judged someone because of their weight.
15. Stared at someone because of their weight.
16. Blamed something on someone because of their weight.
17. Called someone a name because of their weight.
18. Suggested a diet to someone because of their weight.
19. Nagged someone to lose weight.
20. Had low expectations about someone because of their weight.
21. Laughed at someone because of their weight.
22. Teased someone because of their weight.
23. Grimaced at someone because of their weight.
