# Supplementary material for: Lack of guilt, shame, and remorse following weight stigma expression: a real-time assessment pilot study
Source: PeerJ. 2020 Dec 22;8:e10294. doi: 10.7717/peerj.10294 (PMC7761191; doi:10.7717/peerj.10294)
Supplement: Table S1 — Note. Wt. Stig. Weight Stigma. Significant (p <.05) estimates of the linear, quadratic, and cubic components of the trajectories appear in bold. *indicates a trend (<.10) toward statistical significance. [file peerj-08-10294-s002.docx]

Supplementary Table 1. *General estimating equation (GEE) analyses for SSGS guilt, shame, and pride subscales following a weight stigma event.*

|  | Guilt | | | Shame | | | Pride | | |
| --- | --- | --- | --- | --- | --- | --- | --- | --- | --- |
|  | *B* | *SE* | *p* | *B* | *SE* | *p* | *B* | *SE* | *p* |
| Intercept | **1.99** | **.0830** | **<.001** | **2.027** | **.071** | **<.001** | **2.764** | **.037** | **<.001** |
| Hours *  Wt. Stig.  Event  (linear) | **-.247** | **.090** | **.006** | -.096 | .049 | .052* | -.059 | .043 | .166 |
| Hours^2^ *  Wt. Stig.  Event  (quadratic) | .020 | .015 | .167 | .009 | .005 | .077 | .007 | .009 | .412 |
| Hours^3^ *  Wt. Stig.  Event  (cubic) | **-.002** | **<.001** | **.009** | -.001 | <.001 | .225 | <.001 | <.001 | .479 |

*Note. Wt. Stig.* Weight Stigma. Significant (*p* < .05) estimates of the linear, quadratic, and cubic components of the trajectories appear in bold. *indicates a trend (< .10) toward statistical significance
