# Supplementary material for: Lack of guilt, shame, and remorse following weight stigma expression: a real-time assessment pilot study
Source: PeerJ. 2020 Dec 22;8:e10294. doi: 10.7717/peerj.10294 (PMC7761191; doi:10.7717/peerj.10294)
Supplement: Table S2 — Note. Wt. Stig. Weight Stigma. Significant (p < .05) estimates of the linear, quadratic, and cubic components of the trajectories appear in bold. [file peerj-08-10294-s003.docx]

Supplementary Table 2. *General estimating equation (GEE) analyses for the positive affect and negative affect PANAS subscales following a weight stigma event.*

|  | Positive Affect | | | Negative Affect | | |
| --- | --- | --- | --- | --- | --- | --- |
|  | *B* | *SE* | *p* | *B* | *SE* | *p* |
| Intercept | **2.659** | **.0396** | **<.001** | **2.014** | **.073** | **<.001** |
| Hours *  Wt. Stig.  Event  (linear) | **-.089** | **.035** | **.010** | **-.216** | **.080** | **.007** |
| Hours^2^ *  Wt. Stig.  Event  (quadratic) | -.009 | .009 | .314 | .012 | .010 | .243 |
| Hours^3^ *  Wt. Stig.  Event  (cubic) | <.001 | <.001 | .828 | -.001 | <.001 | .060 |

*Note. Wt. Stig.* Weight Stigma. Significant (*p* < .05) estimates of the linear, quadratic, and cubic components of the trajectories appear in bold.
